# Improving carbohydrate and starch accumulation in *Chlorella* sp. AE10 by a novel two-stage process with cell dilution

**DOI:** 10.1186/s13068-017-0753-9

**Published:** 2017-03-24

**Authors:** Dujia Cheng, Dengjin Li, Yizhong Yuan, Lin Zhou, Xuyang Li, Tong Wu, Liang Wang, Quanyu Zhao, Wei Wei, Yuhan Sun

**Affiliations:** 1 Shanghai Advanced Research Institute, Chinese Academy of Sciences, 99 Haike Road, Shanghai, 201210 China; 2 University of Chinese Academy of Sciences, 19 Yuquan Road, Beijing, 100049 China; 3 ShanghaiTech University, 100 Haike Road, Shanghai, 201210 China

**Keywords:** *Chlorella*, Carbohydrate, Starch, Light intensity, Nitrogen starvation

## Abstract

**Background:**

Microalgae are highly efficient cellular factories that capture CO_2_ and are also alternative feedstock for biofuel production. Carbohydrates, proteins, and lipids are major biochemical components in microalgae. Carbohydrates or starch in microalgae are possible substrates in yeast fermentation for biofuel production. The carbon partitioning in microalgae could be regulated through environmental stresses, such as high concentration of CO_2_, high light intensity, and nitrogen starvation conditions. It is essential to obtain carbohydrate-rich microalgae via an optimal bioprocess strategy.

**Results:**

The carbohydrate accumulation in a CO_2_ tolerance strain, *Chlorella* sp. AE10, was investigated with a two-stage process. The CO_2_ concentration, light intensity, and initial nitrogen concentration were changed drastically in both stages. During the first stage, it was cultivated over 3 days under 1% CO_2_, a photon flux of 100 μmol m^−2^ s^−1^, and 1.5 g L^−1^ NaNO_3_. It was cultivated under 10% CO_2_, 1000 μmol m^−2^ s^−1^, and 0.375 g L^−1^ NaNO_3_ during the second stage. In addition, two operation modes were compared. At the beginning of the second stage of mode 2, cells were diluted to 0.1 g L^−1^ and there was no cell dilution in mode 1. The total carbohydrate productivity of mode 2 was increased about 42% compared with that of mode 1. The highest total carbohydrate content and the highest starch content of mode 2 were 77.6% (DW) and 60.3% (DW) at day 5, respectively. The starch productivity was 0.311 g L^−1^ day^−1^ and the total carbohydrate productivity was 0.421 g L^−1^ day^−1^ in 6 days.

**Conclusions:**

In this study, a novel two-stage process was proposed for improving carbohydrate and starch accumulation in *Chlorella* sp. AE10. Despite cell dilution at the beginning of the second stage, environmental stress conditions of high concentration of CO_2_, high light intensity, and limited nitrogen concentration at the second stage were critical for carbohydrate and starch accumulation. Although the cells were diluted, the growths were not inhibited and the carbohydrate productivity was improved. These results were helpful to establish an integrated approach from CO_2_ capture to biofuel production by microalgae.

## Background

Increasing greenhouse gases emission leads to serious environmental problems. Carbon dioxide is produced during the exploitation, transport, and utilization of fossil fuels. Integration of chemical and biological processes is beneficial to establish alternative green routes for reducing CO_2_ emission [1]. Microalgae are highly efficient cellular factories that capture CO_2_ and produce lots of available products. They are feedstock for the further chemical conversions including catalytic deoxygenation, hydrothermal liquefaction, and pyrolysis processes [2]. Carbohydrates, proteins, and lipids are major biochemical components in microalgae. Hydrocarbons could be converted from oil-rich microalgae [3]. Microalgae accumulate large amounts of carbohydrates through photosynthesis. The accumulated carbohydrate is stored as starch in cells [4]. Carbohydrate-rich microalgae are also possible to be feedstock for green chemical conversions. Ethylene glycol and 1,2-propanediol were directly converted from *Chlorococcum* sp. by a Ni–MgO–ZnO catalyst [5]. The product was 5-hydroxymethylfurfural for same microalgae strain if the commercial H-ZSM-5 zeolite was applied in one-pot conversion [6]. The carbohydrates in microalgae are also possible substrate for bioethanol production by yeast fermentation [7, 8]. Therefore, it is very important to obtain carbohydrate or starch-rich microalgae as feedstock for the next chemical or biochemical conversions [9].

In general, total carbohydrate content in microalgae is about 20% dry weight (DW) and starch content is about 10% DW [10]. Carbohydrate and starch contents vary with microalgae species, cultivation conditions and cultivation time [3, 11]. *Chlorella* sp. is widely utilized for CO_2_ capture, wastewater treatment, and development of high value-added products. Cultivation conditions including nutrient starvation, light intensity, light–dark cycle, and CO_2_ concentration could regulate the carbon partitioning in the cells to carbohydrates and lipids [12]. Light intensity, light–dark cycle, and CO_2_ concentration are related to photosynthesis which is a critical step for carbohydrate and starch biosynthesis. A previous study focused on the effects of high light intensity (600 μmol m^−2^ s^−1^) and light–dark cycle on starch and lipid synthesis in six *Chlorella* species [13]. The starch content was higher under 2% CO_2_ compared to that in air at the first 6 days [14]. A slight increase of carbohydrate content was also identified among three *Chlorella* strains when the CO_2_ concentration was increased from 1 to 30% [15]. A high concentration of CO_2_ promoted carbohydrate formation in some microalgae strains while it may inhibit their growth [16]. Besides light intensity, cell cycle is also an essential parameter for increasing starch content in *Chlorella* [4]. It is widely recognized that nitrogen starvation leads to lipid accumulation in some green microalgae. Limited nitrogen concentration is a usual environmental stress to improve lipid productivity [17, 18]. However, previous study on *Isochrysis zhangjiangensis* (Haptophyta) showed that carbohydrates, rather than lipids, accumulated rapidly under nitrogen-depleted conditions [19]. Sulfur starvation was also applied for enhancing starch production [20]. Multiple stress conditions were used for improving carbohydrate production, for example, carbohydrate, or starch-rich microalgae were obtained under both high light intensity and nitrogen starvation conditions [21–26]. A two-stage process is an ordinary method to improve lipid or carbohydrate accumulation in microalgae [4, 22, 26]. Abundant biomass was produced in the first stage while the lipid or carbohydrate was accumulated under stress conditions at the second stage.

High concentration of carbohydrate or starch in microalgae is beneficial for chemical conversion and fermentation. High carbohydrate or starch productivity is favorable to obtain much more feedstock. Both carbohydrate content and carbohydrate productivity are important for the next applications. The aim of the current study is to propose an optimal bioprocess strategy for carbohydrate accumulation in microalgae under high light intensity, high concentration of CO_2_ and nitrogen deficiency conditions. Some studies focused on carbohydrate accumulation while others proposed experimental data about starch content and its productivity. The starch content was also evaluated which will be compared with the reported experimental data in references.

## Results

### Effect of culture mode on biomass and carbohydrate productivities

**Fig. 1:**
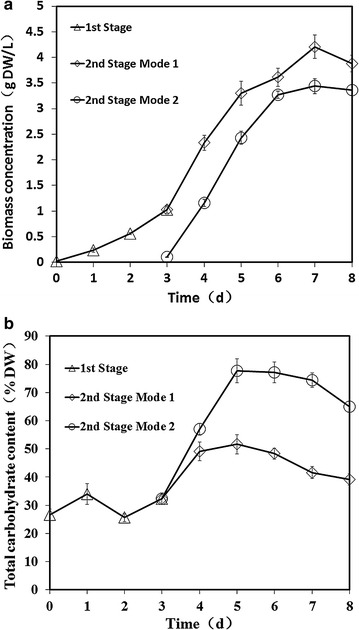
Comparison of two modes of cultivation. Growth profile (**a**) and total carbohydrate content in DW (**b**) of *Chlorella* sp. AE10 under two modes of cultivation

**Fig. 2:**
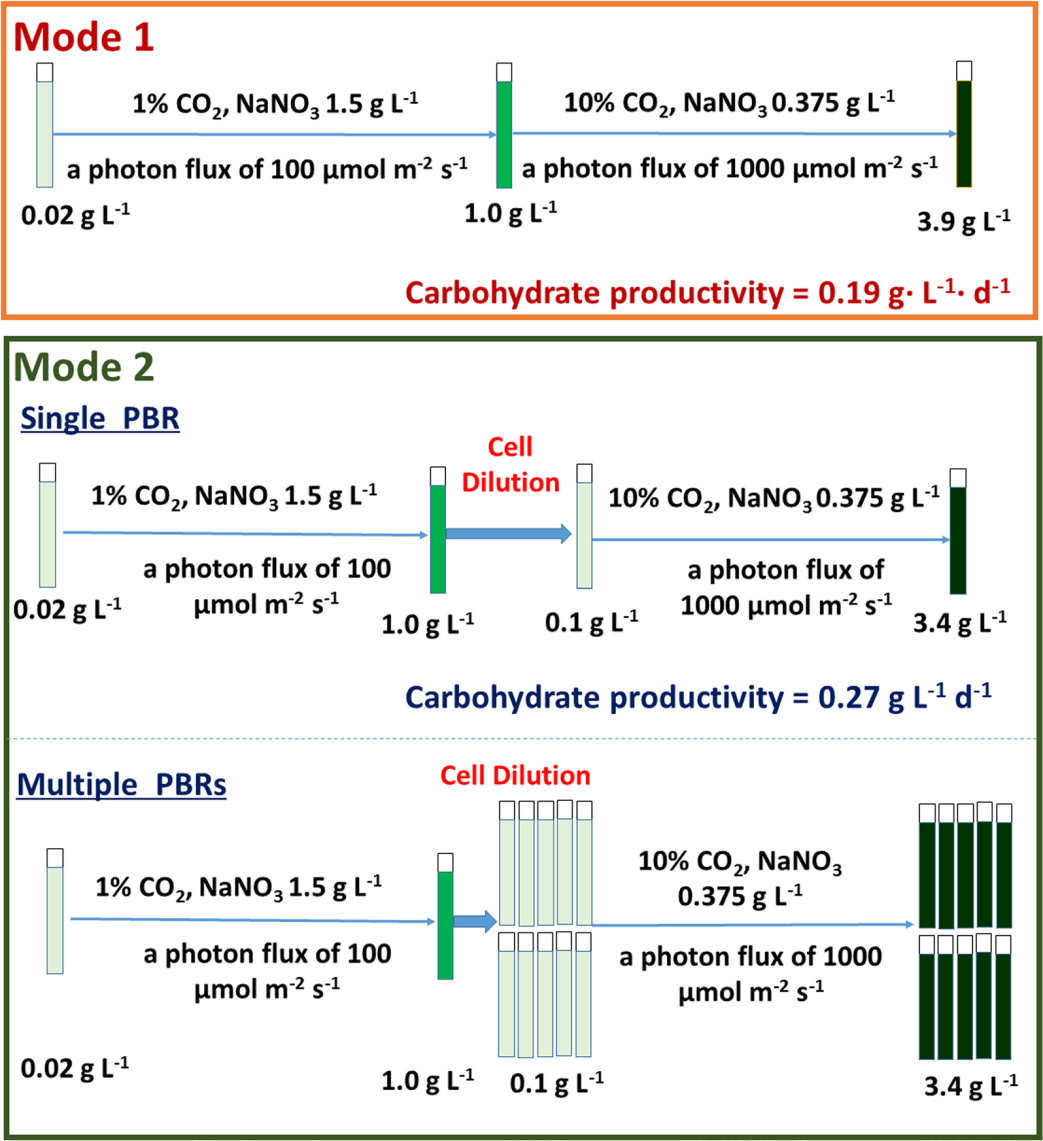
Comparison of two modes for carbohydrate production. *Mode 1* is without cell dilution and *mode 2* is with cell dilution. The first stage of both modes was from day 0 to day 3. The second stage was from cell dilution to day 8. Data were from Fig. 1

Mode 1 consisted of an ordinary two-stage cultivation. The final biomass concentration of mode 1 at day 8 was about 3.9 g L^−1^ which was higher than that of mode 2 as shown in Fig. 1. The maximum total carbohydrate content of mode 1 was 51.6% (DW) but that was near to 77.6% (DW) of mode 2 at day 5. The new established two-stage process improved carbohydrate productivity in a single PBR. The maximum biomass productivity of mode 2 was 0.546 g DW L^−1^ day^−1^ and carbohydrate productivity was greater than 0.421 g DW L^−1^ day^−1^ (at day 5). The final carbohydrate productivity of mode 1 was 0.19 g DW L^−1^ day^−1^ while that of mode 2 was 0.27 g DW L^−1^ day^−1^ in a single PBR during the entire cultivation (a totally of 8 days). It should be mentioned that the cells can be diluted into 10 PBRs in mode 2. Although the final carbohydrate productivity of each one of the ten PBRs was same to a single PBR, 7.65 g (DW) carbohydrate could be obtained in mode 2 but that was 0.53 g (DW) in mode 1. This new mode is effective to harvest much more microalgal biomass and carbohydrate as shown in Fig. 2.

### Effects of initial cell density in the second stage

**Fig. 3:**
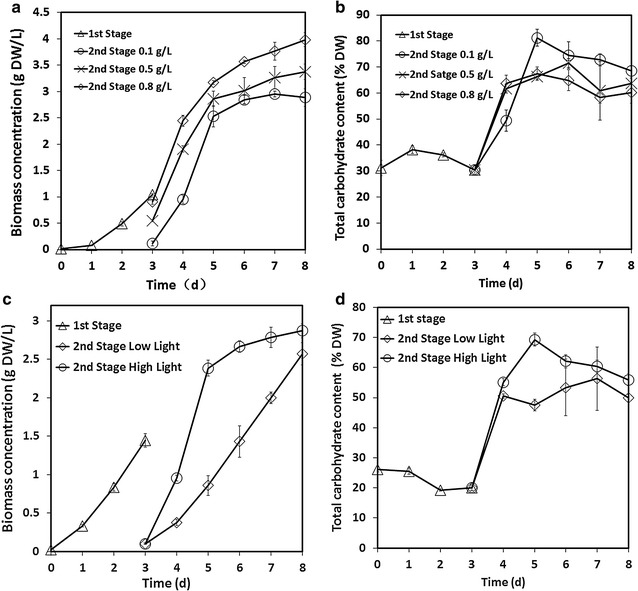
Effects of initial cell density and light intensity. Growth profile (**a**) and total carbohydrate content (**b**) of *Chlorella* sp. AE10 when the initial cell density at the second stage was 0.1, 0.5, and 0.8 g L^−1^; growth profile (**c**) and total carbohydrate content in DW (**d**) of *Chlorella* sp. AE10 under high and low light intensity. From day 1 to day 3, it was cultivated under 100 μmol m^−2^ s^−1^. From day 4 to day 7, it was cultivated under 1000 or 100 μmol m^−2^ s^−1^. The initial cell density at the second stage was 0.1 g L^−1^ for experiments of light intensity (**c**, **d**)

In this study, cell dilution was performed at the beginning of the second stage which was different to other two-stage processes [22, 26]. The selected stress conditions led to high carbohydrate and biomass productivities although the initial cell density was very low as shown in Fig. 3a, b. At the end of the first stage, the cell density was about 1 g L^−1^. When the initial cell density at the second stage was increased from 0.1 to 0.8 g L^−1^, the related final biomass concentration at day 8 increased from 3 to 4 g L^−1^. The maximum growth rate of approximately 0.927 g L^−1^ day^−1^ was obtained from day 3 to day 5 when the initial cell density at the second stage was 0.1 g L^−1^. It is relevant to note that the harvested cells at the end of the first stage could be diluted into 10 PBRs if the initial cell density at the second stage was 0.1 g L^−1^. It could be diluted into 2 PBRs when the initial cell density at the second stage was 0.5 g L^−1^. Although the final carbohydrate productivities under three initial cell densities were similar in a single PBR, the obtained total carbohydrate content after the whole process was significantly higher than those of the other initial cell densities when the initial cell density at the second stage was 0.1 g L^−1^. In previous experiment (data not shown), the growth rate was very low if the initial cell density was lower than 0.1 g L^−1^ at the second stage. For the cultivation of *Chlorella fusca* under 1000 μmol m^−2^ s^−1^, the initial cell density was 1.0 g L^−1^ [25]. Higher initial cell density could tolerate the environmental stress but it was not a good choice for improving carbohydrate productivity of *Chlorella* sp. AE10.

### Effects of light intensity in the second stage

**Fig. 4:**
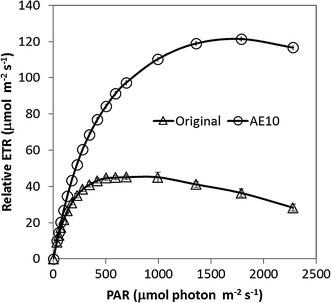
Rapid light curve. RLC for *Chlorella* sp. AE10 and its original strain without adaptive evolution (*ETR* electron transport rate, *PAR* photosynthetic active radiation)

Carbon dioxide concentration and light intensity, which are essential for photosynthesis, can greatly affect the carbohydrate accumulation in microalgae. The biomass concentration and carbohydrate content under both light intensities are shown in Fig. 3c, d. In general, high light intensity (more than 300 μmol m^−2^ s^−1^) leads to reactive oxygen species so it is not suitable for most of the species of microalgae growth [13]. In this study, however, biomass accumulation increased under higher light intensity compared with microalgae grown under low light intensity. The final biomass concentration was about 2.8 g L^−1^ cultivated under high light intensity conditions which was 11.7% higher than that of *Chlorella* sp. AE10 cultivated under low light intensity conditions at day 8. Compared with the original strain without adaptive evolution, *Chlorella* sp. AE10 could tolerate higher light intensity from 500 to 1800 μmol m^−2^ s^−1^ and the efficiency of photosynthesis increased more than one time as shown in Fig. 4. The carbohydrate content increased significantly during the first 2 days of the second stage when *Chlorella* sp. The highest total carbohydrate content was 70% under high light intensity at day 5. It was shown that high light intensity in the second phase was very important to have a higher carbohydrate productivity of *Chlorella* sp. AE10. The high total carbohydrate content indicates efficient photosynthesis.

**Fig. 5:**
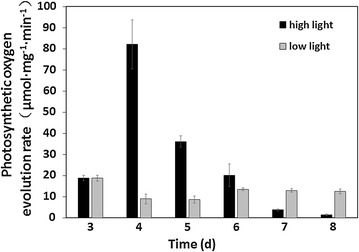
Photosynthetic oxygen evolution rates. They were determined during the second stage under low light intensity (100 μmol m^−2^ s^−1^) and high light intensity (1000 μmol m^−2^ s^−1^)

The oxygen evolution rate is an accurate characterization method of photosynthetic rate. The photosynthetic oxygen evolution rate was measured daily in the second stage. As shown in Fig. 5, when *Chlorella* sp. AE10 was transferred to a high light intensity condition, the photosynthetic oxygen evolution rate increased. The highest photosynthetic oxygen evolution rate was detected on day 4, which is 4 times to that on day 3. The photosynthesis rate of *Chlorella* sp. AE10 decreased gradually under high light intensity conditions. The photosynthetic oxygen evolution rate remained at a higher level under low light intensity conditions. Although *Chlorella* sp. AE10 could tolerate a high light intensity, the photosynthetic oxygen evolution rate tended to be zero at day 8 under 1000 μmol m^−2^ s^−1^. It was indicated that the released oxygen in photosynthesis of *Chlorella* sp. AE10 was close to the absorbed oxygen in respiration at this time.

### Effects of CO_2_ concentration in the second stage

**Fig. 6:**
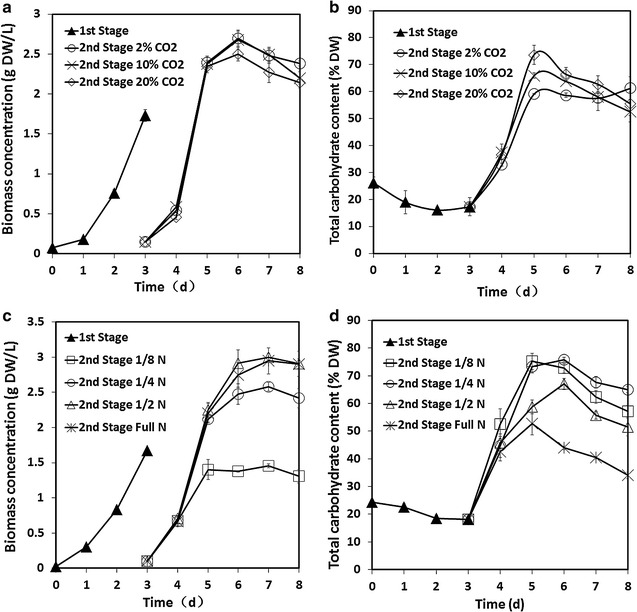
Effects of CO_2_ concentration and initial NaNO_3_ concentrations. Growth profile (**a**) and total carbohydrate content in DW (**b**) of *Chlorella* sp. AE10 under different CO_2_ concentrations (2, 10 and 20%) at the second stage; growth profile (**c**) and total carbohydrate content in DW (**d**) of *Chlorella* sp. AE10 under different initial NaNO_3_ concentrations (1/8, 1/4, 1/2 N, and full N; full N, 1.5 g L^−1^) at the second stage

The *Chlorella* sp. AE10 could tolerate 10–30% CO_2_ (v/v) and its carbohydrate content was in the range of 40–50% [15]. The optimal CO_2_ concentration in the second stage was investigated. The growth profile and total carbohydrate content are shown in Fig. 6a, b. Consistent with a previous study, the growth rate of *Chlorella* sp. AE10 under 20% CO_2_ was a little lower than that under 1% or 10% CO_2_ from day 6 to day 8. The total carbohydrate content was increased when the CO_2_ concentration increased at day 5. The highest carbohydrate productivity was obtained under 10% CO_2_ in the second stage which was similar to the CO_2_ concentration of industrial flue gas.

### Effects of initial nitrogen concentration in the second stage

As shown in Fig. 6c, d, the biomass concentration and the total carbohydrate content fluctuated drastically based on the initial nitrogen concentrations. A maximum biomass, 3 g L^−1^, was obtained in full nitrogen medium. However, it is interesting to note that the total carbohydrate content had the opposite performance on the full nitrogen medium. The highest total carbohydrate content was more than 70% at day 5 and day 6 in 1/4 nitrogen medium. The limited nitrogen concentration from 1/4 N to 1/2 N could support the microalgae growth during the second stage. Although the total carbohydrate content was also about 70%, the biomass concentration was only 1.5 g L^−1^ when the initial nitrogen concentration was 1/8 N. Nitrogen-free media was also performed at the second stage while cells were died at day 5 (data not shown).

### Starch biosynthesis and its metabolic regulation

**Fig. 7:**
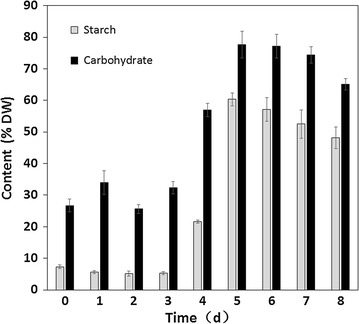
Starch and total carbohydrate contents in DW of *Chlorella* sp. AE10 under two modes

**Fig. 8:**
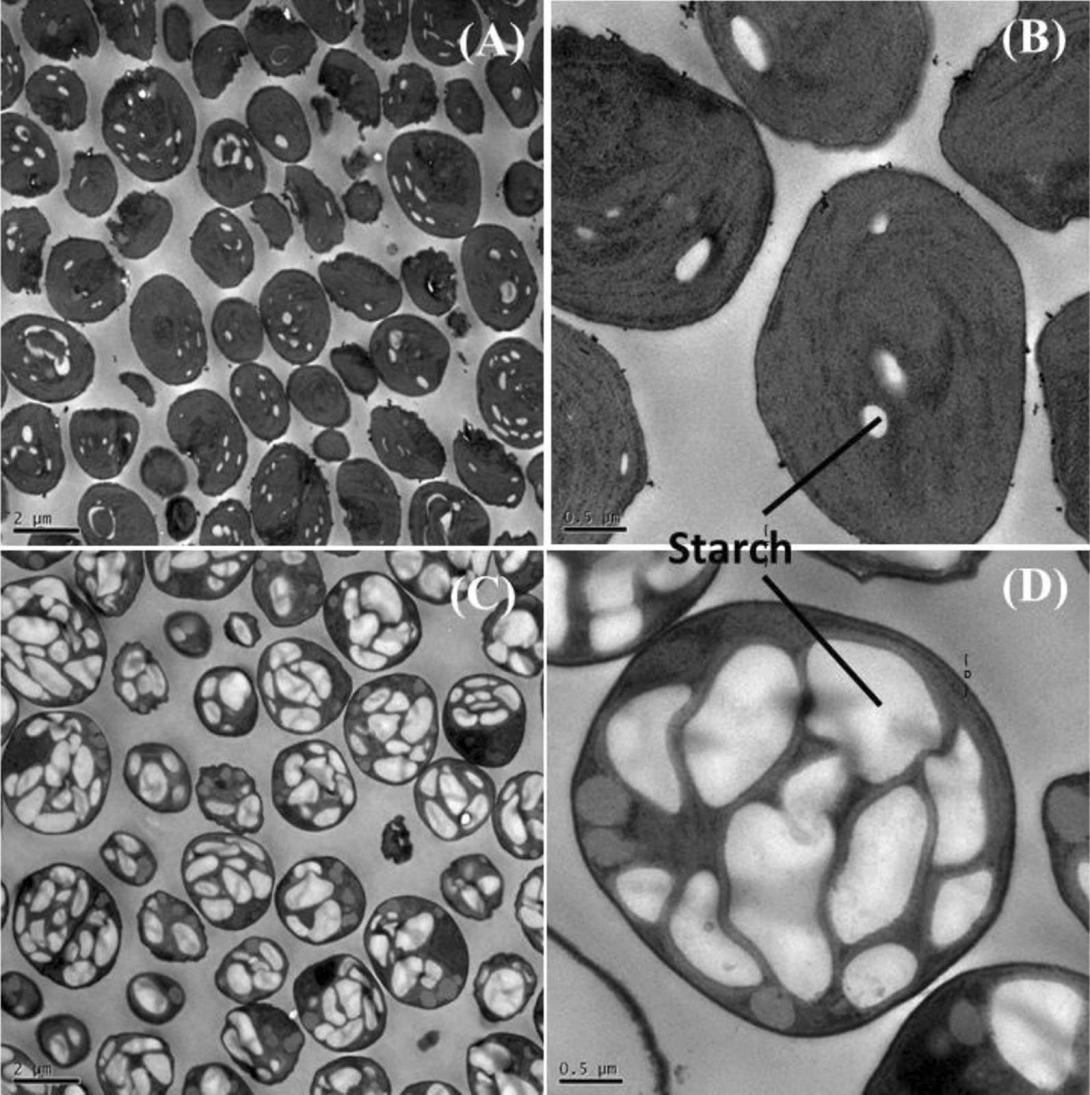
TEM images of *Chlorella* sp. AE10. Cellular structure of *Chlorella* sp. AE10 at day 0 (**A**, **B**) and day 5 (**C**, **D**): **A** bar = 2 μm; **B** bar = 0.5 μm; **C** bar = 2 μm; **D** bar = 0.5 μm

The carbon fixation takes place during photosynthetic reactions. The carbon partitioning is essential for the development of biofuels and chemicals. In general, most carbohydrates are stored as starch in microalgae. In order to explore metabolic regulation mechanism in the second stage, the starch content was characterized and multiple gene expressions related to CO_2_ fixation, carbohydrate synthesis, and lipid metabolism were determined. When the total carbohydrate content increased from 32.4% (day 3) to 77.6% (day 5), the related starch content was 5.3% (day 3) and 60.3% (day 5) as shown in Fig. 7, respectively. The significant change was also visible in related TEM micrographs as shown in Fig. 8. More starch accumulated in microalgae which was consistent with the results of starch content analysis. Additionally, both the maximum carbohydrate productivity (0.421 g L^−1^) and the maximum starch productivity (0.311 g L^−1^ day^−1^) were obtained from day 0 to day 6. It means that 6 days are enough for carbohydrate accumulation in the two-stage process.

**Fig. 9:**
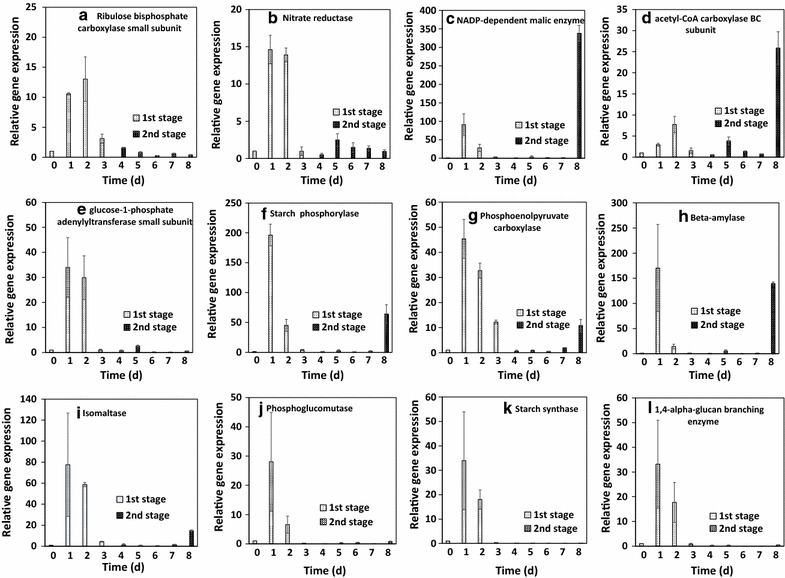
Relative mRNA levels of 12 enzymes during the two-stage process. **a** rbcS; **b** NR; **c** ME; **d** accC; **e** AGPase; **f** SP; **g** PEPcase; **h** BAM; **i** ISA; **j** PGM; **k** SS; **l** AGB

**Fig. 10:**
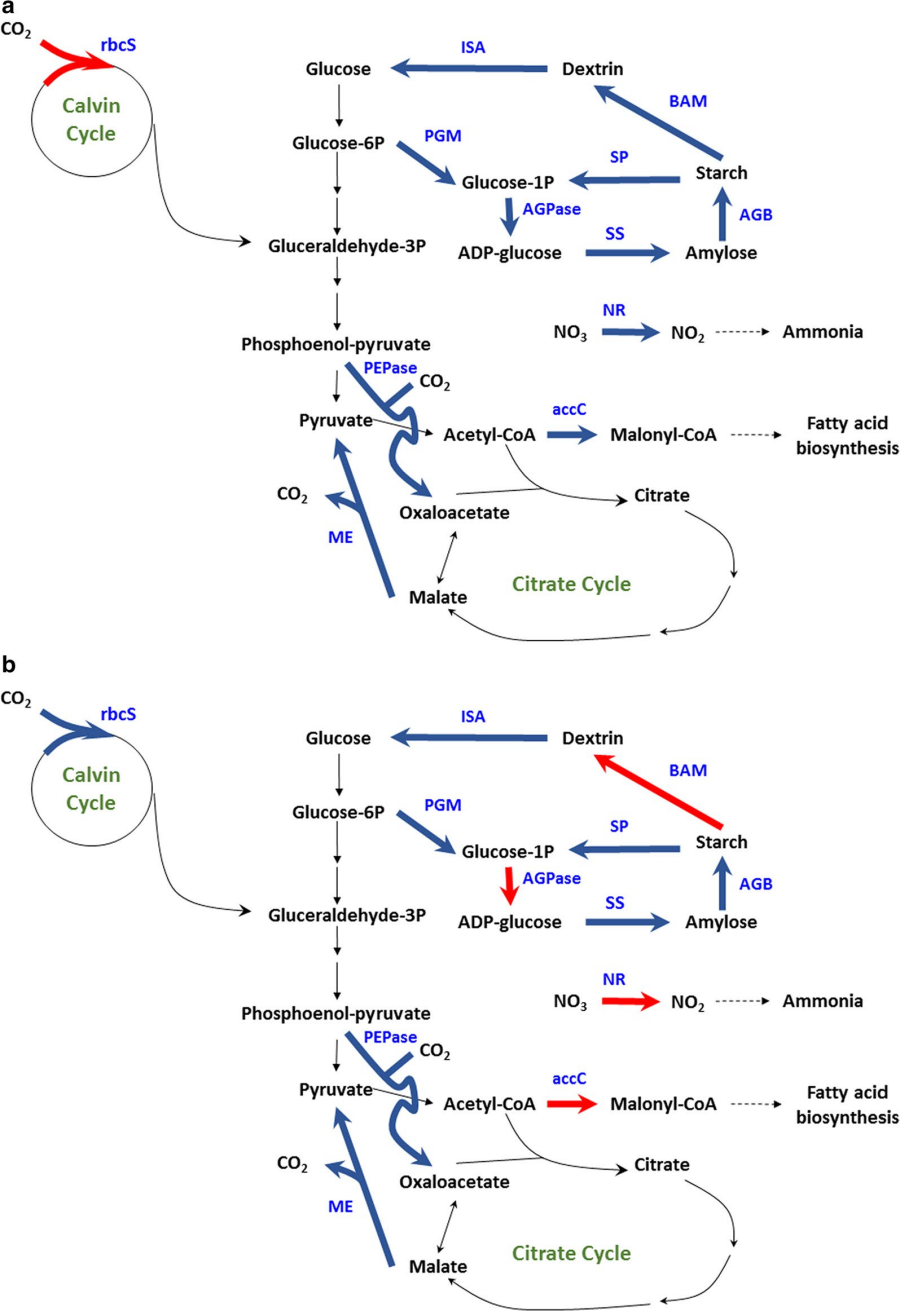
Metabolic regulation for CO_2_ fixation and starch accumulation at day 4 (**a**), day 5 (**b**). The gene expression patterns were shown in *bold lines*. The *red* means over-expressed and the *blue* means down-regulated

**Fig. 11:**
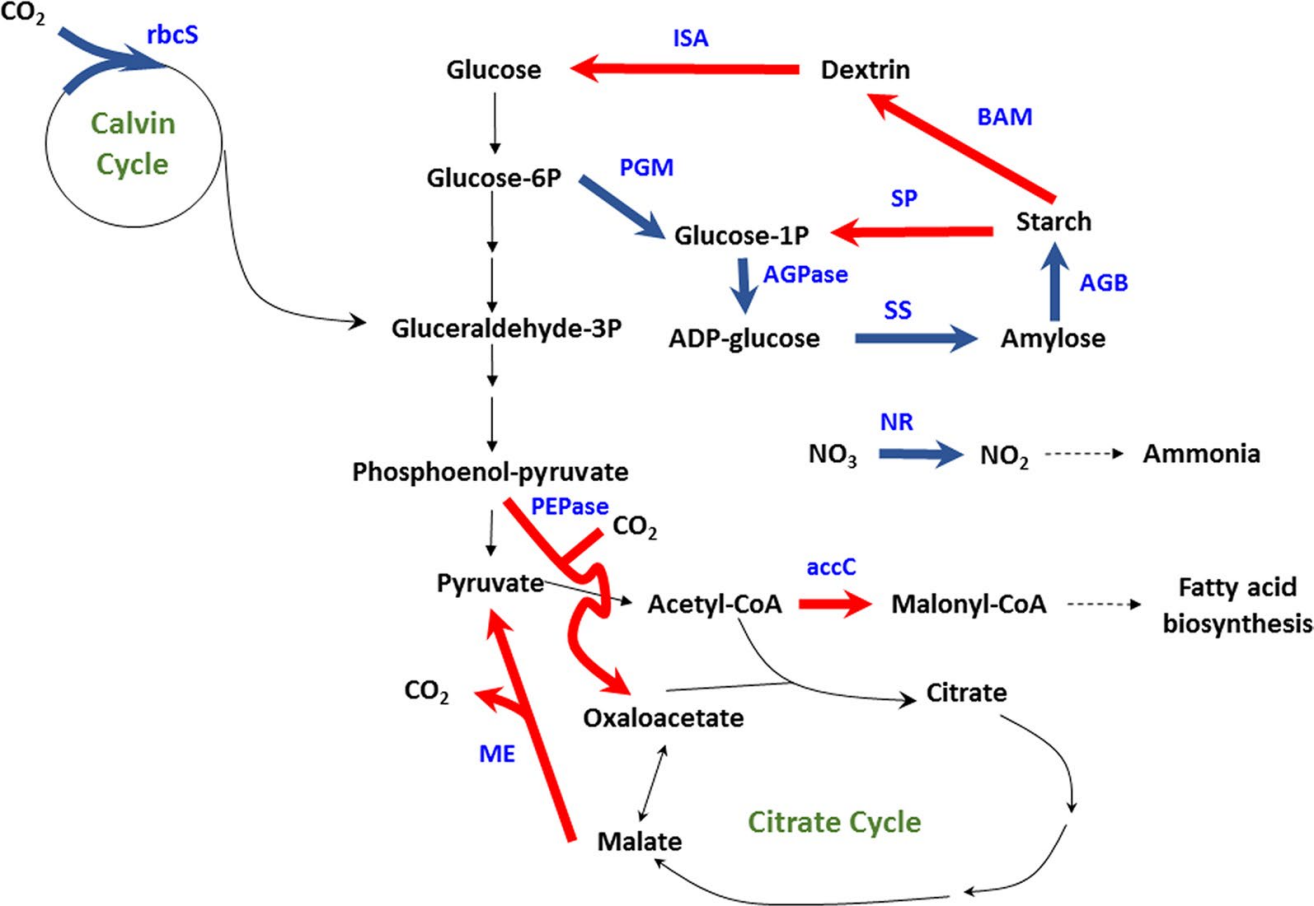
Metabolic regulation for CO_2_ fixation and starch accumulation at day 8 . The gene expression patterns were shown in *bold lines*. The *red* means over-expressed and the *blue* means down-regulated

The relative mRNA expression of 12 enzymes in photosynthesis, CO_2_ fixation, lipid metabolism, and starch metabolism are shown in Fig. 9. At day 1 and day 2, most of them were more than 10. At this time, microalgae were under rapid growth state. After day 4, they were cultivated under stress conditions. Most of them were very low from day 4 to day 7. It is consistent with the results of photosynthetic oxygen evolution rate as shown in Fig. 5. Carbohydrates and starch were accumulated during this period. It is interesting that the related mRNAs were highly expressed for ME, accC, SP, PEPCase, BAM, and ISA (Fig. 9c, d, f–i) at day 8. It is indicated that the starch was degraded and lipid content was increased in microalgae which was also proved for *C. sorokiniana* [27], *S. obliquus* [28], and *C. zofingiensis* [29]. The possible mechanism of carbohydrate or starch accumulation is shown in Figs. 10 and 11. At the day 4, most of the investigated genes were down-regulated while the gene related to ribulose bisphosphate carboxylase small unit was up-regulated (Fig. 10a). It was possible to enhance the CO_2_ fixation by Calvin cycle and it was related to the increase of biomass concentration at day 4. The gene related to glucose-1-phosphate adenylyltransferase small unit was up-regulated at day 5 meant that it was very important for starch accumulation (Fig. 10b). At day 8, the genes related to starch degradation and fatty acid biosynthesis were up-regulated as shown in Fig. 11. It was indicated that the lipid was increased and the starch concentration was reduced from that time.

## Discussion

The carbohydrate and starch productivities determined by this study were compared with experimental data in references as shown in Table 1. In this study, the maximum total carbohydrate content was higher than other results in references and its starch content was similar to that of marine microalgae, *T. subcordiformis* [20]. During the first 2 days of the second stage, carbohydrate and starch were accumulated rapidly. The carbohydrate and starch productivity were 0.921 and 0.730 g L^−1^ day^−1^, respectively at the second day of the second stage. In general, nitrogen starvation, and other stress conditions are only used to regulate the carbon partitioning while they could not support the rapid growth of microalgae. The stress conditions integrated with high light intensity, high concentration of CO_2_, and limited nitrogen concentration at the second stage led to high total carbohydrate content from day 4 to day 5. It regulated the carbon partitioning into carbohydrate and maintained a stable level. Similarly to this study, 8 days cultivations were performed for *S. obliquus* CNW-N [24] and *N. oleoabundans* HK-129 [26]. Five or six days were selected for the first stage to obtain enough biomass of both microalgae. In general, high cell density is helpful to tolerate environmental stresses. Cell dilution was made in this study to enhance the effects of high light intensity, high concentration of CO_2_, and nitrogen limited conditions. *Chlorella* sp. AE10 has higher tolerance to high concentration of CO_2_, high light intensity and nitrogen deficiency. Three days was conducted at the first stage and a cell dilution was performed at the beginning of the second stage while similar carbohydrate productivity was obtained under these conditions. It was indicated that *Chlorella* sp. AE10 had a strong potential for producing feedstock of biofuels and chemicals.

**Table 1:** Comparison of performances for *Chlorella* sp. AE10 and other strains reported in the references

Microalgae strain	Light intensity (μmol m^−2^ s^−1^)	CO_2_ (%, v/v)	N or S condition	Maximum total carbohydrate content (% DW)	Carbohydrate productivity (g L^−1^ day^−1^)	Maximum starch content (% DW)	Starch productivity (g L^−1^ day^−1^)	Ref.
*Scenedesmus obliquus* CNW-N	240	2.5	−N	52.88	0.468^a^	–	–	[24]
*Neochloris oleoabundans* HK-129	200	4	−N	41.30	0.047	–	–	[26]
*Scenedesmus obliquus* CNW-N	140	2.5	−N	46.70	0.383	–	–	[22]
*Chlorella zofingiensis*	150	1	−N	66.9	0.407	43.4	0.268	[12]
*Tetraselmis subcordiformis*	200	3	−S	–	–	62.1	0.62	[20]
*Chlorella fusca*	1200	1.5	−N	–	–	49.0	0.38	[25]
*Chlorella* sp. AE10	1000	10	0.375 g L^−1^ NaNO_3_	77.6	0.421^b^	60.5	0.311^b^	This study
					0.927^c^		0.730^c^	

^a^Semi-batch system

^b^The carbohydrate or starch productivity is calculated with experimental data from day 0 to day 6

^c^The carbohydrate or starch productivity is calculated with experimental data from day 3 to day 5

Although the two-stage process with cell dilution could obtain much more algal biomass in theory, it also needs much more photobioreactors, operation cost, and water for cultivation. The technical economy is dependent on all of these conditions.

## Conclusions

In this study, a novel two-stage process was evaluated for improving carbohydrate and starch accumulation of the microalgae *Chlorella* sp. AE10. The highest carbohydrate content was 77.6% and the highest starch content was 60.3%. The starch productivity was 0.311 g L^−1^ day^−1^ and the carbohydrate productivity was 0.421 g L^−1^ day^−1^ from day 0 to day 6. The carbohydrate productivity of *Chlorella* sp. AE10 in mode 2 was increased about 42% compared with that of mode 1. *Chlorella* sp. AE10 could tolerate high concentration of CO_2_ and high light intensity. It was important to maintain rapid growth under these environmental stress conditions. Cell dilution at the second stage also led to high carbohydrate and starch productivities. These results will be helpful to establish an integrated approach from CO_2_ capture to biofuel production by microalgae.

## Methods

### Experimental organism and cultivation conditions

*Chlorella* sp. AE10 was obtained after a long period of adaptive laboratory evolution under 10% CO_2_ [15]. It was maintained in BG11 medium. All experiments were carried out in a tube photobioreactors (PBRs) maintained at a temperature of 28 ± 0.05 °C by a constant temperature circulating water bath. The tubes were cylindrical with a height of 40 cm and diameter of 4.5 cm. Its working volume was 350 ml. Input gas was 0.1 L min^−1^ of CO_2_-enriched air. All cultivations were performed under continuous illumination.

### Optimization of culture conditions at the second stage

A two-stage process was performed to improve carbohydrate productivity. The two modes for this two-stage process are detailed in Fig. 2. Mode 1 was a continuous cultivation without cell dilution at the beginning of the second stage. The microalgal cells were diluted after 3 days in mode 2. All other conditions of mode 1 and mode 2 were same. In the first stage (from day 0 to day 3), microalgae were cultivated under 1% CO_2_, 100 μmol m^−2^ s^−1^, and 1.5 g L^−1^ NaNO_3_ (initial concentration). In the second stage (after cell dilution until day 8), stress conditions were conducted to improve the carbohydrate productivity. In order to investigate the influence of these cultivation conditions at the second stage of mode 2 on improving biomass and carbohydrate productivities, inoculation density, CO_2_ concentration, light intensity, cultivation mode, and initial nitrogen concentration were evaluated in the experiments. Inoculation cell density was varied at the concentrations, 0.1, 0.5, or 0.8 g DW L^−1^. Three CO_2_ concentrations, 1, 10, and 20%, were selected for the CO_2_ concentration gradient. White LEDs were used throughout the cultivations from one side of the PBRs. The average light intensity was around 100 or 1000 μmol m^−2^ s^−1^ measured by a LI-250 Light Meter with a quantum sensor (SR.NO.Q49770 of QUANTUM, LICOR, USA) inner the tube PBR. The modified BG11 media with different initial nitrogen concentrations, 0, 0.19 g L^−1^ (1/8 N), 0.38 g L^−1^ (1/4 N), 0.75 g L^−1^ (1/2 N), and 1.5 g L^−1^ (full N) NaNO_3_, were applied for investigating the effects of nitrogen concentration on improving high carbohydrate content in the second stage. Biomass and polysaccharide content was measured daily.

### Test for photosynthetic oxygen evolution rate

Two milliliter of sample collected daily was used to determine photosynthetic oxygen evolution rate after 20 min of dark adaptation. Every sample was measured 8 min using Oxy-Lab (Hansatech, UK) with a set of magnetic rotor speed of 300 r min^−1^ and the light intensity was same to the experimental conditions during cultivations. Take the average slope of eight times as the photosynthetic oxygen evolution rate of the sample. Rapid light curve (RLC) [30] was determined for *Chlorella* sp. AE10 and the original strain without adaptive evolution by Fluorescence Monitoring System (Hansatech Instruments, UK).

### Determination of biomass concentration

Biomass concentration was determined gravimetrically from 10 ml samples daily collected by suction filtration. The cells were filtrated with a microfiltration membrane (φ50 × 0.45 μm,CN-CA) and weighed by analytical balance after drying 24 h in a thermostatic drier box (105 °C, DHG-9070A). Biomass concentration was expressed as dry weight (DW) per liter.

### Analysis of total carbohydrate content

Total carbohydrate content was measured by phenol–sulfuric acid method [15]. About 0.1 ml of sample was added to a 2 ml tube. Then 0.1 ml of 5% phenol (v/v) solution was added and mixed. Next, 0.5 ml of concentrated sulfuric acid was added rapidly and mixed well by vortexing. After cooling 30 min at room temperature to allow color development, 200 μl of the solution was pipetted into the bottom of a 96-well microplate (Greiner Bio-One, 655101) and the absorbance was measured at 490 nm with iMark™ microplate reader (BIO-RAD). Another 0.1 ml of sample was centrifuged at 13,400 rpm for 10 min and the absorbance of the supernatant was also determined using the method described above. The standard curve was made with determined values of different concentrations of glucose standard solutions. The total carbohydrate content was calculated by determined values of solution and supernatant.

### Analysis of starch content

The starch content of samples collected daily was determined using Megazyme Total Starch kits (K-TSTA, Ireland). The samples collected at day 0 and day 5 were used for observation of morphology by transmission electron microscopy (TEM, JEM-1230). Additional details regarding this method were reported in the Ref. [31]. Photos were taken using a Gatan Orius SC 200w camera.

### cDNA synthesis and real time-PCR analysis

RNA was extracted daily during the two-stage cultivation. Microalgae cultivated under 1% CO_2_, 100 μmol m^−2^ s^−1^ and 1.5 g L^−1^ NaNO_3_ (initial concentration) were used as control. All the RNA samples were extracted by Trizol Plant (Transgene, China), and cDNA was synthesized using High Capacity RNA to cDNA Kit (Transgene, China). The cDNAs from each sample were used for quantitative PCR analysis using Power SYBR Green Real Time qPCR Master Mix (Transgene, China). Target genes were amplified on a StepOne-Plus (Applied Biosystems, Spain) for rbcS (ribulose bisphosphate carboxylase small subunit), NR (nitrate reductase), ME (NADP-dependent malic enzyme), accC (acetyl-CoA carboxylase BC subunit), AGPase (glucose-1-phosphate adenylyltransferase small subunit), SP (starch phosphorylase), PEPCase (phosphoenolpyruvate carboxylase), BAM (beta-amylase), ISA (isomaltase), PGM (phosphoglucomutase), SS (starch synthase), AGB (1,4-alpha- glucan branching enzyme), and the housekeeping gene 18S rRNA. The primers shown in Table 2 were synthesized by Shanghai Sunny biotechnology Co., Ltd. The threshold cycle (Ct) values from triplicate reactions were averaged and logarithmically transformed. Delta Ct values (ΔCt) were calculated using 18S rRNA, and delta delta Ct (ΔΔCt) values relative to the determined expression level [32].

**Table 2:** Primers in this study for gene expression analysis

Gene or enzyme	EC number	Primer pairs (5′–3′)	Ref.
18S rRNA	–	F: ATCAACCTGACAAGGCAACC	[27]
		R: CCTGCGGCTTAATTTGACTC	
*rbcS*	4.1.1.39	F: CTGCGTACACCAGCAACG	[33]
		R: CCCAGTACCGGTTGTCGTA	
*NR*	1.7.1.1	F: TACTACCACTTCCACGACAACC	[33]
		R: TACCACCAGCCCTCCTTGT	
*ME*	1.1.1.40	F: CTGCAGGCATGTCCAGTG	[33]
		R: AGACCCAGGATGCGTTCTC	
*accC*	6.4.1.2	F: CAATCATGATCAAGGCTACCG	[33]
		R: AGGAACTCGCTCTCGTCCT	
*AGPase*	2.7.7.27	F: AACTCCACCTCGCTCAACC	[33]
		R: CCAGCACCTCCACAAAGC	
*SP*	2.4.1.1	F: AGCCTGTATCTGCAGGTTGC	[33]
		R: TTGCAGGTTGGTTGTTTGG	
*PEPCase*	4.1.1.31	F: GTTAATCTTTGCTATCATGCAACC	[33]
		R: GCTCCTCCAGGATGTGCTC	
*BAM*	3.2.1.2	F: GTACCCGTCCTACCCAGAGG	[27]
		R: TGTCGTAGCACTGGAACTGG	
*ISA*	3.2.1.10	F: CCACCGCCTCTGTCAACT	[27]
		R: CGTTGGCCTCATTGTGCT	
*PGM*	5.4.2.2	F: GACTCCAACATTGCGAAGAT	This study
		R: GGAAGAGGTCGGTCAGGT	
*SS*	2.4.1.21	F: GCCTGGTCAACCTGTGGAT	This study
		R: GAACACCTGCCGCATCAC	
*AGB*	2.4.1.18	F: TGATGTGCTTCCTCACCCT	This study
		R: TCCAAAGTGTCCTGGTCCT	

### Statistical analysis

Results were shown as means of three biological replicates and the error bars indicated the standard deviation. The statistical significance of the gene expressions was evaluated using Student’s *T* Test. In all cases, comparisons were considered as significant one if *P* value was less than 0.05.

## Abbreviations

rbcS: ribulose bisphosphate carboxylase small subunit
NR: nitrate reductase
ME: NADP-dependent malic enzyme
accC: acetyl-CoA carboxylase BC subunit
AGPase: glucose-1-phosphate adenylyltransferase small subunit
SP: starch phosphorylase
PEPCase: phosphoenolpyruvate carboxylase
BAM: beta-amylase
ISA: isomaltase
PGM: phosphoglucomutase
SS: starch synthase
AGB: 1,4-alpha- glucan branching enzyme
RLC: rapid light curve
